# The challenges of translation

**DOI:** 10.15252/emmm.201910874

**Published:** 2019-10-18

**Authors:** Helmut R Salih, Gundram Jung

**Affiliations:** ^1^ Clinical Collaboration Unit Translational Immunology German Cancer Consortium (DKTK) Department of Internal Medicine University of Tübingen Tübingen Germany; ^2^ Department of Immunology Institute for Cell Biology University of Tübingen Tübingen Germany; ^3^ German Cancer Consortium (DKTK) DKFZ partner site Tübingen Tübingen Germany

**Keywords:** Cancer, Immunology

## Abstract

Cancer immunotherapy is a highly active area in translational medicine where the challenges and rewards of developing new drugs “from bench to bedside” become particularly visible. Here, we comment on both, the scientific and non‐scientific hurdles of this translational process using the example of bispecific antibodies (bsAbs) and chimeric antigen receptor (CAR) T cells, two closely related strategies for antibody‐guided recruitment of T cells against cancer. Both exert impressive therapeutic activity and were recently approved for treatment of B‐cell malignancies. We discuss how the efficacy of these auspicious therapeutic tools may be further improved, in particular against solid tumors, but we also address another critical issue: Since both approaches were already introduced in the 1980s, why did it take almost thirty years until they became clinically available?

## Graphical abstract

Cancer immunotherapy offers formidable opportunities for patients. In this Commentary, H. Salih and G. Jung discuss the scientific and societal challenges to overcome to accelerate the development of new drugs and the transition from bench to bedside.

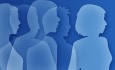

Thirty years ago, one of us (GJ), a young physician at that time, was attending a meeting entitled “Targeted Cellular Cytotoxicity and Bispecific Antibodies” in Annapolis, MA, USA, organized among others by David Segal. Four years earlier, Segal and his colleagues (Perez *et al*, 1985), and Uwe Staerz together with Mike Bevan (Staerz *et al*, 1985) had introduced the concept of bsAbs comprising a target and a TCR/CD3 specificity to induce T‐cell reactivity against any desired target cell. GJ suggested to combine these reagents with bsAbs stimulating the costimulatory T‐cell molecule CD28 (Jung *et al*, 1987), which has remained a focus of his work ever since. Ten years later, another young physician (HRS) became attracted to immunotherapy in general and bsAbs in particular and joined GJ, who at that time conducted a clinical study with CD28‐stimulating bsAbs produced in his laboratory to treat glioblastoma patients (Jung *et al*, 2001). Back at the Annapolis meeting, another speaker, Zelig Eshhar, introduced T cells transfected with chimeric receptors comprising antibody‐binding parts (CAR T cells; Gross *et al*, 1989). The results he reported were quite similar to those achieved with bsAbs: antibody‐guided killing of tumor cells by T cells irrespective of MHC restriction.

The 1990s saw the generation of the first recombinant bsAbs in a single‐chain format, later known as BiTE (bispecific T‐cell engager). In the following years, the prototypical CD19xCD3 antibody blinatumomab was established for treatment of B cell‐derived leukemias and lymphomas. However, optimal dosing of this bsAb was and still is complicated owing to unspecific and potentially life‐threatening cytokine release, which limits safely applicable doses and, in turn, efficacy. In addition, the low serum half‐life of the drug requires cumbersome continuous infusion. Despite these limitations, blinatumomab shows impressive therapeutic activity and was eventually approved in 2014 (Riethmuller, 2012).

During the 1990s, single‐chain antibodies likewise were used for generating CAR T cells, but the therapeutic activity of these constructs was limited. This changed dramatically when the signaling units of costimulatory molecules, such as CD28 and 4‐1BB/CD137, were introduced into the CAR constructs. The importance of co‐stimulation reflects physiological T‐cell activation, where costimulatory signaling is required for sustained activity. The resulting second‐generation CAR T cells are impressively successful against CD19‐expressing leukemia. First clinical approval of CAR T cells was obtained in 2017 and thus, similar to the bsAb blinatumomab, almost 30 years after conceptualization (Pang *et al*, 2018). Notably, CAR T cells and bsAbs share other similarities: Both reagents cause cytokine release syndrome (CRS), a systemic inflammatory response, as a consequence of unwanted T‐cell activation, and they come at prices that oncologists had not thought possible a decade ago—in the case of CAR T cells up to US$ 300,000 per treatment.

## Scientific challenges

Bispecific antibodies and CAR T cells have contributed to the breakthrough of immunotherapy and expand the therapeutic armamentarium of oncologists. Where are we heading with these promising but also challenging approaches? At present, their activity appears to be less impressive against solid tumors when compared to hematological malignancies. In our view this is, at least in great part, owing to the insufficient accessibility of solid tumor sites for T cells: Even large numbers of tumor‐specific T cells fail to exert sufficient antitumor activity without a proinflammatory environment at the tumor site (Ganss *et al*, 2002). This critical limitation may be overcome by targeting antigens expressed on both, tumor cells as well as tumor vessels, the latter allowing for influx of effector cells across a damaged endothelial barrier. However, antigens suitable for such “dual targeting” are scarce. Possible candidates are the prostate‐specific membrane antigen (PSMA) and the more recently identified CD276 (B7H3) molecule, a member of the immunomodulatory B7 family. We have developed a PSMAxCD3 bsAb designated CC‐1. This reagent comprises a novel PSMA binder that allows for such dual targeting in prostate carcinoma and notably also in squamous cell lung cancer. Our bsAb was constructed in a novel IgG‐based, Fc‐attenuated format (IgGsc) with increased serum half‐life and reduced off‐target T‐cell stimulation. Good manufacturing practice (GMP) production and clinical evaluation of CC‐1 were entirely financed by public money provided by the Helmholtz foundation and the German Cancer Consortium (DKTK); the clinical trial will start recruiting in late 2019 (NCT04104607). In addition, we are currently evaluating optimized bsAbs with CD276‐specificity. Notably, both PSMA and CD276 have also been used as targets for CAR T cells by other investigators; results of clinical studies with these dual targeting concepts will hopefully soon become available.

Beyond the so far limited efficacy against solid tumors, there are other drawbacks of both bsAbs and CAR T cells that need to be addressed, particularly the undesired sequelae of cytokine release and T‐cell exhaustion after excessive stimulation. We think that these challenges can be overcome by prophylactic rather than symptom‐triggered IL‐6 blockade and by optimized strategies for co‐stimulation, respectively (Long *et al*, 2015).

## Societal challenges

As explained above, most technical challenges were successfully resolved during the 1990s. Why then did it take so long for bsAbs and CAR T cells to arrive where they are today? In our opinion, the central problem is neither of scientific nor technical nature: It is the ever‐increasing regulatory burden with regard to GMP (good manufacturing practice) production and clinical evaluation based on the so‐called “good clinical practice” (GCP). These hurdles drastically prolong the time from conceptualization of a drug to its clinical evaluation and generate high costs. The expenses for GMP‐compliant production of a bsAb, for example, may easily reach US$ 5 million; the first studies in patients have a similar price tag. Usually, funding at such a level is not available at public institutions, which are thereby in effect barred from drug development. This is somewhat ironic, since it has been such institutions where the concepts of bsAbs and CAR T cells were initially developed and clinically evaluated.

Do patients benefit from the excessive regulatory requirements? GMP solely reduces the technical, but not the biological risk of a drug, as the latter is related to its mechanism of action. This is exemplified by the deleterious “TeGenero incident” involving a “superagonistic” CD28 antibody that induced life‐threatening CRS in six healthy volunteers (Hunig, 2012). Since the side effects were caused by the biological function of the drug, they were not prevented by the utterly applied regulatory procedures.

Most importantly, when discussing risks and safety, we must not ignore that the biggest threat for a cancer patient confronted with failing conventional treatment options is the disease itself. To provide such patients access to promising new drugs more rapidly, we suggest to reduce the “GMP requirements”—and thereby costs—in a responsible way, for instance, by adhering to the principle of end‐product rather than in‐process quality control for early clinical evaluation. Likewise, we share the skepticism regarding the increasingly cumbersome, costly and meanwhile almost prohibitive GCP regulation and fully support the initiative of hundreds of colleagues from all over Europe for a new approach (Le Gouill *et al*, 2017).

Ideally, first in man studies should be co‐designed by physician scientists who substantially contributed to the development of a given drug in the first place. Can we be sure that such physicians, guided by a reduced “GMP and GCP formalism”, would design meaningful and safe studies? We believe, yes. Doctors certainly have personal interests, but we trust that their main driving force still is the medical need of their patients and that they best know the strengths and risks of their own product. What we have in mind is a revival of the classical role of the physician scientist that goes far beyond being an effective “GCP doctor” who recruits as many patients as possible for industry‐sponsored trials. We should remember that, for the larger part of the history of modern medicine, physicians not only applied new drugs, but also played a major role in drug development. This holds true for towering figures such as Paul Ehrlich and Emil von Behring who invented the “serotherapy” for diphtheria more than hundred years ago, but also for colleagues like Hans‐Jochem Kolb who more recently developed and applied donor lymphocyte infusion for treatment of leukemia (Kolb *et al*, 1990). This would not have been possible by strictly adhering to nowadays GMP and GCP guidelines, as Kolb has frequently stated.

Will they ever come back then, the old but not outdated times, when not only the pharmaceutical industry but also physicians invented new drugs? We think there is hope, if the burden of GMP and GCP regulation is—thoughtfully and responsibly—reduced for public institutions treating patients confronted with a life‐threatening disease and failing conventional treatment modalities.

Coming to the end of this story, one might ask why the (CD28) co‐stimulating bsAbs, introduced by GJ along the CD3‐stimulating bsAbs and CAR T cells 30 years ago, did so far not make it into the clinic, and whether co‐stimulation would change the bsAb story for the better, as it did for the CAR T cells? Certainly, in this case the TeGenero incident aggravated the challenges discussed above. However, there is reason to believe that the clinical evaluation of bispecific co‐stimulators will not have to wait for better times to come, but to quote the German Novelist Michael Ende: “that's another story and shall be told another time”.

## Conflict of interest

GJ and HRS are listed as inventors on the patent application “Novel PSMA binding antibody and uses thereof”, EP16151281 and others, with the German Cancer Research Center, Heidelberg, Germany, as applicant.
